# Gender-based stigma and the prevention and treatment of HIV/AIDS among older women: A scoping review protocol

**DOI:** 10.1371/journal.pone.0298024

**Published:** 2024-02-08

**Authors:** Thi Vu, Jasmine Manalel, Kate Nyhan, Katie Wang, Joan Monin

**Affiliations:** 1 Department of Social and Behavioral Sciences, School of Public Health, Yale University, New Haven, CT, United States of America; 2 Brookdale Center for Health Aging, Hunter College, City University of New York, New York, NY, United States of America; 3 Cushing/Whitney Medical Library, Yale University, New Haven, CT, United States of America; Edith Cowan University, AUSTRALIA

## Abstract

**Introduction:**

The population of women aged 50 years and older living with HIV is increasing. Older women face unique challenges in the prevention and management of HIV; however, they are often under engaged in HIV/AIDS research. One such challenge is gender-based stigma, which can be manifested through harmful gendered stereotypes, discrimination, prejudice, and sexism that could potentially hinder HIV care engagement among this population. We propose a scoping review to identify and synthesize evidence pertaining to how experiences of gender-based stigma impacts HIV prevention and care among older women.

**Materials and methods:**

We will use the framework by Arksey and O’Malley and the Preferred Reporting Items for Systematic Reviews and Meta-Analyses extension for scoping reviews (PRISMA-ScR) to conduct this scoping review. We will search MEDLINE/PubMed, Web of Science, PsycINFO, CINAHL and Scopus for empirical literature published between January 1981 and the date of search commencement. Supplementary screening will be conducted using backwards citation chaining of the final list of included full-text articles. Two reviewers will independently screen all titles and abstracts for articles that meet the predetermined inclusion criteria. Two reviewers will also screen full-text articles and chart data using a standardized data collection form.

**Results:**

We will synthesize the findings through tables, charts, and narrative summaries. We will also identify gaps in the current literature and provide recommendations for future research. Findings will be shared at conferences and submitted to a peer-reviewed publication.

**Discussion:**

To our knowledge, this will be the first scoping review to examine gender-based stigma in relation to HIV prevention and care among older women. We anticipate that our results will be of interest to older women living with HIV, healthcare providers, policy makers, and community activists working to improve quality of life and care experiences for older women living with HIV.

## Introduction

Since the mid-1990s, antiretroviral therapy treatment has been effective for suppressing HIV (human immune-deficiency virus) viral load, leading to a decline in mortality caused by HIV [1]. Currently, over half of the 1.2 million persons living with HIV in the United States (U.S) are aged 50 or older [2]. Much of the HIV-related literature has focused on younger populations and men [3]. However, older women living with HIV (OWLH) represent an increasing proportion of individuals diagnosed with HIV in the U.S [4]. Data from the Centers for Disease Control and Prevention indicates that there are over 100,000 OWLH [5].

A previous systematic review of psychosocial factors related to healthy aging among OWLH identified that the experiences of HIV among older women are different from those of younger women and older men due to compounding effects of intersectional systems of oppression attributed one’s gender, age, race/ethnicity, socioeconomic status, and sexual/gender identity [6,7]. Regarding HIV prevention and awareness, the CDC recommends routine HIV testing for adults ages 13–64. However, older women are less interested in HIV testing, less likely to have been tested in the past, and reported lower perceived HIV risk and knowledge compared to younger women [8,9]. Ageism from health care providers and the belief that older women are either monogamous or not sexually active contribute to lower HIV testing behaviors and higher prevalence of late diagnoses among this population [8,10]. Stereotypes about aging and presumption of the sexual practices of older women may limit them from accessing necessary medical, social, and educational support to address HIV [9,10].

Relationship factors should also be considered in the prevention and care of OWLH. Social support has been consistently correlated with better quality of life and mental health among PLWH by buffering the impacts of HIV stigma [9,11–13]. Older adults not linked to care and social services, or who have limited social networks may experience social isolation and exclusion. One study of over 160 older adults living with HIV found that 79% of respondents lived alone. They also reported unmet instrumental and emotional support needs [14]. Specifically, OWLH may not have access to traditional caregiving support networks, such as children or spouses, who are able and willing to provide daily assistance [15]. Conversely, OWLH may have additional caregiving responsibilities, such as caring for adult children, grandchildren, elderly parents, and partners [3]. Being an informal caregiver for a loved one has been traditionally seen as a woman’s role with 70–75% of informal caregivers being women [15]. For OWLH who are already burdened by HIV-related factors such as side effects of treatment and societal stigma, additional responsibilities can add additional financial, physical, emotional, and mental strain [6]. One study, for example, found that women who had caregiving responsibilities had 1.8 times the odds of delaying care compared to men [15]. Additionally, up to 55% of older women experience intimate partner violence (IPV) [16]. Women living with HIV are at increased risk for IPV. Conversely, women who experience IPV also have higher odds of developing HIV [17,18]. Differential power dynamics in the romantic relationships of OWLH, combined with cultural norms surrounding gender roles, may limit older women from discussing and negotiating safe sex with their partners [10,18].

For OWLH, being diagnosed with HIV can be especially stigmatizing and isolating due to their invisibility relative to other populations in the discussion about HIV (e.g., men who have sex with men, adolescents, people who use intravenous drugs) [10]. There is a dearth of HIV clinical trials focused on women despite the fact that older women experience unique biological factors related to aging, such as menopause and increased risk for memory loss compared to men [10,19]. Furthermore, sexual health education and community-based support programs for persons living with HIV do not target the unique needs and concerns of older women [6,10,18].

Therefore, OWLH face unique psychosocial challenges with HIV prevention and care. The Health Stigma and Discrimination Framework is useful for examining how stigma related to gender, class, race, and sexual orientation intersects with HIV stigma to affect health outcomes [20]. According to this framework, drivers and facilitators of HIV stigma (e.g., fear of infection, laws criminalizing HIV) lead to stigma “marking” where stigma is applied to individuals with HIV [20]. Intersecting stigmas occur when individuals are “marked” with multiple stigmas. For example, older women living with HIV experience gender-based stigma, HIV stigma, and age stigma simultaneously [20]. This marking manifests in a range of stigma experiences (e.g., social isolation, feelings of shame and guilt, discrimination) and practices (e.g., negative stereotypes and prejudices). These factors ultimately influence outcomes for OWLH (e.g., HIV testing, retention in care, adherence to medication) and practices of institutions/organizations (e.g., criminalization of HIV, lack of legal protections for OWLH who are discriminated against) [20]. The intersecting effects of HIV stigma and gender-related stigma has downstream effects, such has higher HIV incidence, morbidity, mortality, and lower quality of life for OWLH [20]. To our knowledge, no review on gender-based stigma in the context of HIV prevention and care among older women currently exists. Gender-based stigma encompasses stereotyping, prejudice, and discrimination directed towards women and is a result of social devaluation associated with one’s gender [21,22]. Due to the paucity of literature using the term “gender-based stigma”, we will be using the terms sexism and gender discrimination, manifestations of gender-based stigma, interchangeably with “gender-based stigma”. This protocol is reported following the guidance of the Preferred Reporting Items for Systematic Reviews and Meta-Analyses Protocols (PRISMA-P) checklist (see S1 Table) [23,24].

### Study rationale

As the population of OWLH continues to increase, understanding how social and health services can best meet the needs of this population is a public health priority. There are currently no systematic or scoping review syntheses of gender-based stigma in the healthcare experiences of OWLH. Given the paucity of HIV research and programs focused on older women, we are conducting a scoping review to obtain an overview of the existing research, systematize findings, and identify knowledge gaps in the relationship between gender-based stigma and HIV-related health outcomes among OWLH. This review will provide an important basis for understanding how social and health programs can better serve the needs of the increasing population of OWLH.

## Methods and analysis

This scoping review will be conducted in alignment with the best practices guidance of the Joanna Briggs Institute (JBI) and reported following the Preferred Reporting Items for Systematic Review and Meta-Analysis (PRISMA) Extension for Scoping Reviews (PRISMA-ScR) [25,26]. A scoping review uses a systematic approach to synthesize the main concepts, theories, findings, and knowledge gaps related to a particular research field. Unlike a systematic review, a scoping review addresses a broader and more exploratory research question. Due to the limited literature on this topic, the lack of previous synthesis, and the broad issue of gender-based stigma, a scoping review is an appropriate method for this protocol. In the following sections, we report on the five main stages of a scoping review set forth by Arksey and O’Malley: (1) identifying the research question, (2) identifying relevant studies, (3) study selection, (4) charting the data, and (5) collating, summarizing and reporting the results [27].

### Step 1: Identifying the research question

The research questions for this scoping review were developed collaboratively by the research team using the Population Concept Context (PCC) framework [28]. Due to the exploratory nature of this review, our main research questions are: 1) What is known about the experiences of gender discrimination among older women living with HIV? and 2) How do these experiences relate to HIV prevention and treatment? Subsequently, our study objectives are: a) to identify and synthesize existing literature on gender-discrimination (concept) in relation to HIV prevention and care (context) among older women (population), and b) to determine gaps in knowledge and future areas of research pertaining to this topic.

This scoping review protocol has been registered on the Open Science Framework (https://osf.io/3qgef).

### Step 2: Identifying relevant studies

We will systematically search the following databases: MEDLINE/PubMed, Web of Science, PsycINFO, CINAHL and Scopus. An example search strategy in CINAHL is presented in Table 1. Using backward citation chaining, we will search the reference list of included full texts for additional relevant literature.

**Table 1:** Example preliminary pilot search strategy in CINAHL Complete as of 12 July 2023, to be modified as needed for other databases.

Search number	Query	Results
1	(MH "Aged+") OR (MH "Middle Age") OR (MH "Geriatrics+")	1,456,836
2	(TI "aging" OR "aged" OR "middle age*" OR "middle adult*" OR "midlife" OR "old* adult*" OR "geriatr*" OR "geront* OR "elder*" OR "senior*") OR (AB "aging" OR "aged" OR "middle age*" OR "middle adult*" OR "midlife" OR "old* adult*" OR "geriatr*" OR "geront* OR "elder*" OR "senior*")	1,662,194
3	(MH "Women+") OR (MH "Female")	2,234,719
4	(TI "woman" or "women" or "female*") OR (AB "woman" or "women" or "female*")	2,376,987
5	(MH "Human ImmunodeficiencyVirus+") OR (MH "HIV Infections+") OR (MH "Acquired Immunodeficiency Syndrome")	94,642
6	(TI "HIV*" OR "human immunodeficiency virus*" OR "AIDS*" OR "acquired immunodeficiency syndrome*") OR (AB "HIV*" OR "human immunodeficiency virus*" OR "AIDS*" OR "acquired immunodeficiency syndrome*")	153,963
7	(MH "Sexism+") OR (MH "Gender Role+")	12,207
8	(TI (gender* bias OR gender* discriminat* or gender* stigma* or gender* microaggression* or gender*stereotyp* or gender* prejudice* or gender* belie*) OR (sex bias OR sex discriminat* or sex stigma* or sex microaggression* or sex stereotyp* or sex prejudice* or sex belie*) OR ("sexis*" OR "gender* role*" OR "sex role*")) OR (AB (gender* bias OR gender* discriminat* or gender* stigma* or gender* microaggression* or gender*stereotyp* or gender* prejudice* or gender* belie*) OR (sex bias OR sex discriminat* or sex stigma* or sex microaggression* or sex stereotyp* or sex prejudice* or sex belie*) OR ("sexis*" OR "gender* role*" OR "sex role*"))	13,538
9	S1 OR S2	1,668,731
10	S3 OR S4	2,378,020
11	S5 OR S6	164,658
12	S7 or S8	16,203
13	S9 AND S10 AND S11 AND S12	169

### Step 3: Study selection

We will employ an iterative, two-stage screening process with two independent reviewers at each stage. In the first stage, two independent reviewers will screen all titles and abstracts for eligibility based on an a priori inclusion criteria defined using the PCC framework presented in Table 2. At the second stage, the reviewers will read the full texts of the selected titles and abstracts remaining after the first stage to ensure that their content meets inclusion criteria. Conflicting decisions at each stage will be resolved first through a discussion between the two reviewers, and subsequently by a third reviewer, if necessary. We will use Covidence, web-based software, to facilitate screening and study selection.

**Table 2:** PCC framework for identifying key concepts of the scoping review.

PCC Component	Definition used in this study
Population	Eligible studies should include women ages 50 years and older. Studies that also include women younger than 50 years of age and/or men ages 50 years and older should explicitly discuss differences in outcomes by age/gender.
Concept	Eligible studies should explicitly discuss aspects of gender-based stigma, gender discrimination, sexism, harmful gender roles, and/or harmful gender stereotypes. For the purposes of this scoping review, these terms are collectively conceptualized as devaluation, prejudice, and harassment/discrimination based on one’s sex, gender identity, or gender expression.
Context	Eligible studies should discuss gender-based stigma among OWLH in relation to psychosocial, behavioral, and well-being outcomes related to HIV prevention, management, and/or treatment.

#### Eligibility criteria

We will include all empirical research articles employing qualitative, quantitative, or mixed methods. Additionally, articles must: a) be written in English; b) have been published from January 1981 to the date of search commencement; c) have included women ages 50+ in their sample (studies may include younger women and/or older men in addition to older women, but must include explicit discussion of how results differ gender/age); d) explicitly discuss gender discrimination/gender-based stigma/sexism in relation to relevant outcomes. We will be focusing on psychosocial, behavioral, and well-being outcomes in this scoping review [29]. These will include a) mental health (depressive symptoms, emotional and mental distress, anxiety); b) quality of life; c) physical health; d) social support; e) adherence to antiretroviral therapy; f) access to and usage of health/social services and risk behaviors. This review is not limited to any geographic region or setting. We will exclude books, book reviews, editorials, grey literature, review papers, commentaries, and dissertations due to our focus on empirical literature.

### Step 4: Charting the data

Key information about the study will be charted using a data extraction tool. The research team developed the following initial charting variables to categorize the information and identify key themes: author(s), year published, country where study was conducted, journal, sample size and participant characteristics, setting (e.g., hospital, clinic, community), methods (qualitative/quantitative/mixed-methods), how gender-based stigma was measured/conceptualized, health outcome(s) measured, significant findings, funding source, and reported study limitations. To minimize discrepancy in the charting process, we will pilot the extraction tool on a randomly selected 10% sample of the final list of full texts that meet the inclusion criteria. Two reviewers will independently chart this sample of articles and meet to discuss potential inconsistencies in data extraction. We will revise the extraction tool as necessary based on the reviewers’ discussions and ongoing consultation with the team and content experts. We will divide the remaining full texts among the reviewers and continue to employ an iterative, constant comparative method to screen the remaining full texts where we adjust the extraction tool as necessary.

### Step 5: Collating, summarizing, and reporting the results

We will not conduct a formal risk of bias assessment, as this scoping review is only intended to provide an overview of the literature. However, we will discuss any limitations in the methodology and findings of the included studies. A flowchart diagram will be created to document the study selection process. The results of this study will be summarized quantitatively through frequency counts (e.g., to highlight the number of qualitative versus quantitative papers) and qualitatively through thematic analysis (e.g., to describe how OWLH report experiencing gender discrimination). Tables, charts, and narrative summaries are potential strategies we will use to report our findings. We anticipate that our findings will map out the existing knowledge in the relationship between gender-based stigma and HIV-related outcomes among OWLH. Additionally, we will identify gaps in the existing literature and discuss potential areas for future research in the field of aging and HIV pertaining to older women. Due to the potential scope and heterogeneity of the final list of articles, we will refine methods for collating and reporting the results as reviewers become more familiar with the included literature.

## Ethics and dissemination

Since this scoping review is secondary synthesis of existing literature, ethics approval is not required. Study findings will be disseminated to relevant stakeholders, such as OWLH, researchers, health care providers, social workers, and case managers through newsletters, social media, conferences, and manuscripts. Additionally, we will present these findings at national and international conferences, as well as peer-reviewed publications.

### Patient and public involvement

The design of this protocol did not involve patients or the public. However, we aim to engage with support groups within the community who serve OWLH to verify the results of this review.

## Conclusion

The growing population of OWLH in the U.S warrants further exploration into structural factors that impact HIV prevention and management. Understanding how gender-based stigma influences the health of OWLH can help tailor intervention efforts to better meet the needs of women who are aging with HIV. This protocol describes a detailed plan for a scoping review to identify relevant literature regarding how gender-based stigma impacts HIV prevention and care among OWLH. The findings of this review may be limited due to our focus empirical studies published in English from peer-reviewed sources. Additionally, we are not conducting formal quality assessments to determine the strength of the existing research literature. However, our goal for this scoping review is to provide an overview of the current literature on gender-based stigma and HIV among older women. Our protocol will allow us to systematically chart and map the results across multiple databases to identify literature gaps and future areas for research. As such, we expect these findings to be of interest to practitioners, researchers, women aging with HIV, and community advocates working to understanding how to better support OWLH in their healthcare needs.

## Supporting information

S1 Table Preferred Reporting Items for Systematic Reviews and Meta-Analyses Protocols (PRISMA-P) checklist. (DOCX)
